# Correction: Clinical efficacy and safety of interferon (Type I and Type III) therapy in patients with COVID-19: A systematic review and meta-analysis of randomized controlled trials

**DOI:** 10.1371/journal.pone.0296219

**Published:** 2023-12-15

**Authors:** Seungeun Ryoo, Dae-Hyup Koh, Su-Yeon Yu, Miyoung Choi, Kyungmin Huh, Joon-Sup Yeom, Jung Yeon Heo

There are errors in the Funding section. The correct Funding statement is: This research was supported by the National Evidence-based Collaborating Agency, Republic of Korea (grant number: NA22-008 and NA23-010). There was no additional internal or external funding received for this study. The funding source had no role in the study design, data collection, and analysis, decision to publish, or manuscript preparation of the manuscript.

## References

[1] Ryoo S, Koh D-H, Yu S-Y, Choi M, Huh K, Yeom J-S, et al. (2023) Clinical efficacy and safety of interferon (Type I and Type III) therapy in patients with COVID-19: A systematic review and meta-analysis of randomized controlled trials. PLoS ONE 18(3): e0272826. 10.1371/journal.pone.0272826 36989209 PMC10057835

