# Supplementary material for: Clinical efficacy and safety of interferon (Type I and Type III) therapy in patients with COVID-19: A systematic review and meta-analysis of randomized controlled trials
Source: PLoS One. 2023 Mar 29;18(3):e0272826. doi: 10.1371/journal.pone.0272826 (PMC10057835; doi:10.1371/journal.pone.0272826)
Supplement: S1 Table — (PDF) [file pone.0272826.s001.pdf]

S1 Table. Patient severity stratification

Severity	NIAID 8-point Ordinal scale		Hospitalized	Oxygen Therapy/ medical care	IMV	MV or ECMO
	Score	Definition				
1. Uninfected	1	not hospitalized and no limitations of activities	X			
2. Ambulatory (Mild)	2	not hospitalized, with limitation of activities, home oxygen requirement, or both	X			
	3	hospitalized, not requiring supplemental oxygen, and no longer requiring ongoing medical care	O	X/X		
3. Moderate	4	hospitalized, not requiring supplemental oxygen but requiring ongoing medical care	O	X/O		
4. Severe	5	hospitalized, requiring any supplemental oxygen	O	O/O		
	6	hospitalized, requiring noninvasive ventilation or use of high-flow oxygen devices	O		O	
4. Severe / 5. Critical	7	hospitalized, receiving invasive mechanical ventilation (IMV) or extracorporeal membrane oxygenation (ECMO)	O			O
5. Critical	8	Death				
