# Supplementary material for: Clinical efficacy and safety of interferon (Type I and Type III) therapy in patients with COVID-19: A systematic review and meta-analysis of randomized controlled trials
Source: PLoS One. 2023 Mar 29;18(3):e0272826. doi: 10.1371/journal.pone.0272826 (PMC10057835; doi:10.1371/journal.pone.0272826)

S1 Fig. Forest plot of recovery on day 14

Study or Subgroup	IFN Events	IFN Total	SOC or placebo Events	SOC or placebo Total	Weight	Risk Ratio M-H, Random, 95% CI
**1.4.1 Moderate**						
Kalil 2021 (1)	74	84	59	68	23.0%	1.02 [0.90, 1.15]
**Subtotal (95% CI)**		**84**		**68**	**23.0%**	**1.02 [0.90, 1.15]**
Total events	74		59			
Heterogeneity: Not applicable						
Test for overall effect: Z = 0.25 (P = 0.81)						
**1.4.2 Moderate to severe**						
Monk 2021 (2)	21	48	11	50	1.9%	1.99 [1.08, 3.67]
**Subtotal (95% CI)**		**48**		**50**	**1.9%**	**1.99 [1.08, 3.67]**
Total events	21		11			
Heterogeneity: Not applicable						
Test for overall effect: Z = 2.20 (P = 0.03)						
**1.4.3 Severe**						
Bhushan 2021 (3)	112	119	118	123	35.1%	0.98 [0.93, 1.04]
Kalil 2021 (4)	328	403	339	414	33.7%	0.99 [0.93, 1.06]
Pandit 2021 (5)	19	20	13	19	6.3%	1.39 [1.01, 1.92]
**Subtotal (95% CI)**		**542**		**556**	**75.1%**	**1.01 [0.93, 1.09]**
Total events	459		470			
Heterogeneity: Tau² = 0.00; Chi² = 4.53, df = 2 (P = 0.10); I² = 56%						
Test for overall effect: Z = 0.14 (P = 0.89)						
**Total (95% CI)**		**674**		**674**	**100.0%**	**1.03 [0.94, 1.12]**
Total events	554		540			
Heterogeneity: Tau² = 0.00; Chi² = 10.81, df = 4 (P = 0.03); I² = 63%						
Test for overall effect: Z = 0.64 (P = 0.52)						
Test for subgroup differences: Chi² = 4.68, df = 2 (P = 0.10), I² = 57.3%						

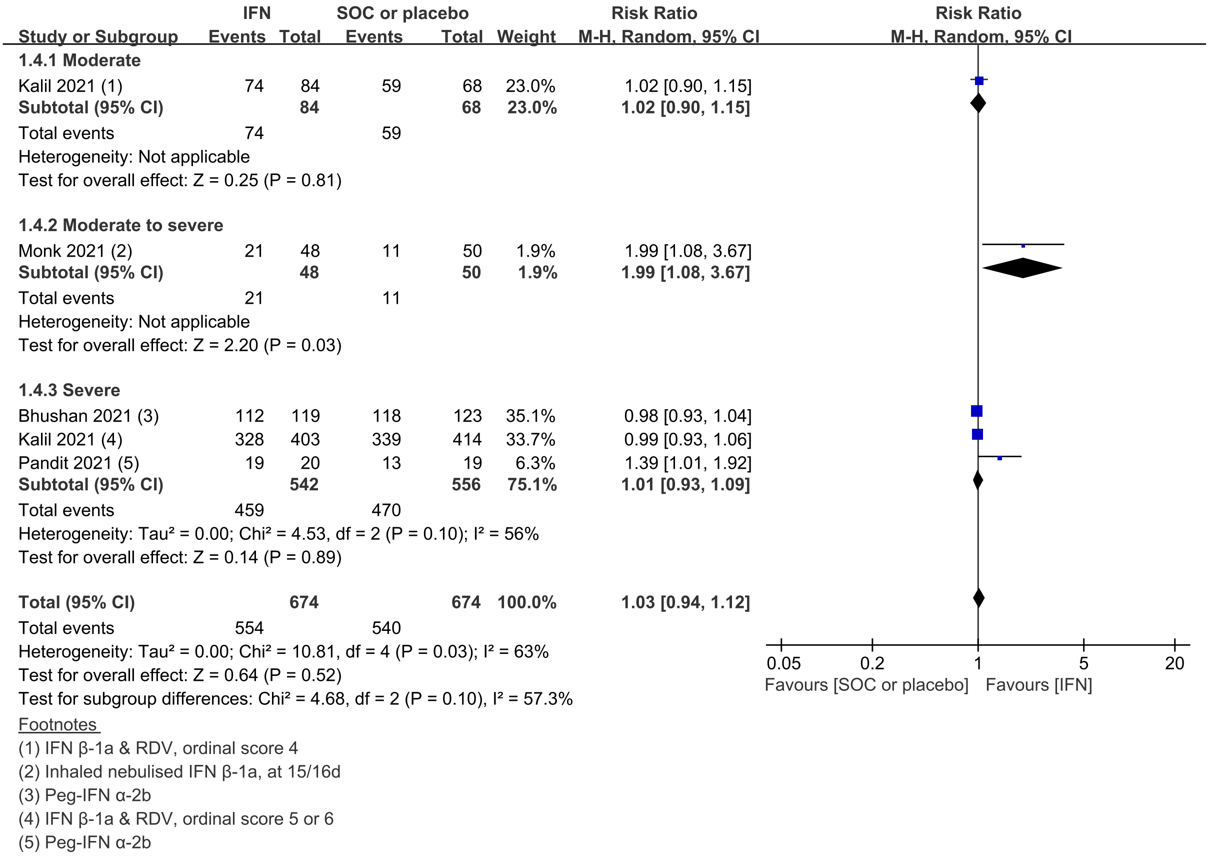

Footnotes
(1) IFN β-1a & RDV, ordinal score 4
(2) Inhaled nebulised IFN β-1a, at 15/16d
(3) Peg-IFN α-2b
(4) IFN β-1a & RDV, ordinal score 5 or 6
(5) Peg-IFN α-2b

S2 Fig. Forest plot of hospital discharge on day 14

Study or Subgroup	IFN Events	IFN Total	SOC or placebo Events	SOC or placebo Total	Weight	Risk Ratio M-H, Random, 95% CI
**1.6.1 Moderate to severe**						
Monk 2021 (1)	35	48	33	50	48.0%	1.10 [0.85, 1.44]
**Subtotal (95% CI)**		**48**		**50**	**48.0%**	**1.10 [0.85, 1.44]**
Total events	35		33			
Heterogeneity: Not applicable						
Test for overall effect: Z = 0.74 (P = 0.46)						
**1.6.2 Severe**						
Davoudi-Monfared 2020 (2)	28	42	17	39	22.6%	1.53 [1.01, 2.32]
Rahmani 2020 (3)	26	33	18	33	29.3%	1.44 [1.01, 2.07]
**Subtotal (95% CI)**		**75**		**72**	**52.0%**	**1.48 [1.13, 1.94]**
Total events	54		35			
Heterogeneity: Tau² = 0.00; Chi² = 0.04, df = 1 (P = 0.84); I² = 0%						
Test for overall effect: Z = 2.83 (P = 0.005)						
**Total (95% CI)**		**123**		**122**	**100.0%**	**1.29 [1.04, 1.59]**
Total events	89		68			
Heterogeneity: Tau² = 0.01; Chi² = 2.40, df = 2 (P = 0.30); I² = 17%						
Test for overall effect: Z = 2.34 (P = 0.02)						
Test for subgroup differences: Chi² = 2.30, df = 1 (P = 0.13), I² = 56.5%						

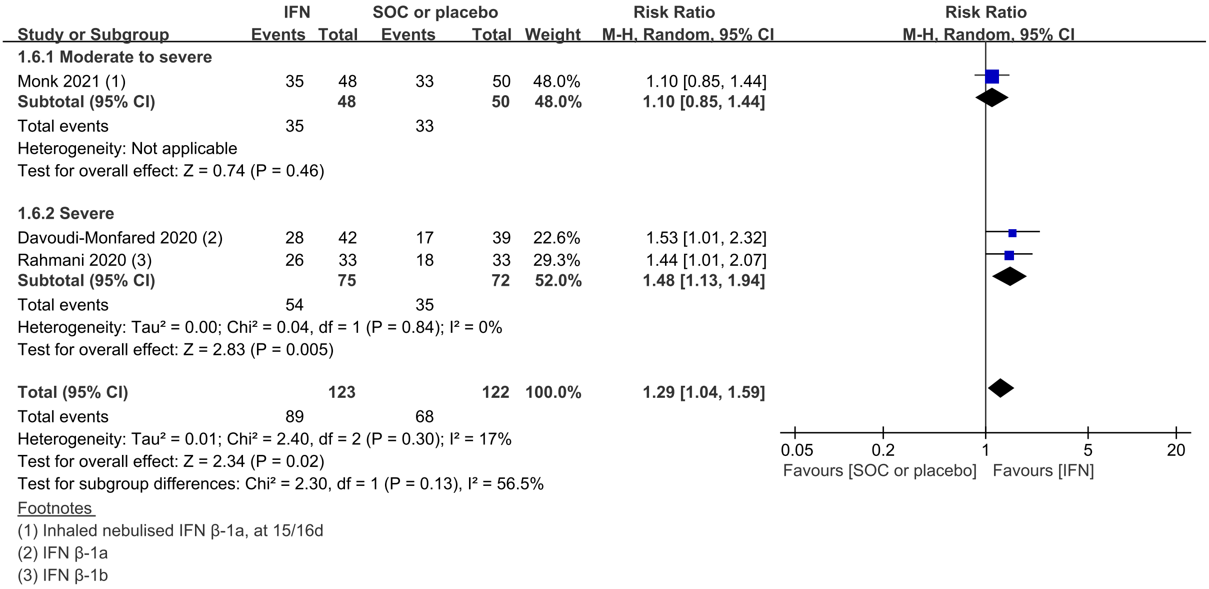

Footnotes

(1) Inhaled nebulised IFN β-1a, at 15/16d

(2) IFN β-1a

(3) IFN β-1b

S3 Fig. Forest plot of time to clinical response

Study or Subgroup	IFN Mean	IFN SD	IFN Total	SOC or placebo Mean	SOC or placebo SD	SOC or placebo Total	Weight	Mean Difference IV, Random, 95% CI
**1.5.1 Mild**								
Jagannathan 2021 (1)	8	9.88	60	9	11.86	60	3.3%	-1.00 [-4.91, 2.91]
**Subtotal (95% CI)**			**60**			**60**	**3.3%**	**-1.00 [-4.91, 2.91]**
Heterogeneity: Not applicable								
Test for overall effect: Z = 0.50 (P = 0.62)								
**1.5.2 Moderate**								
Kalil 2021 (2)	4	2.34	84	4	4.21	68	17.2%	0.00 [-1.12, 1.12]
**Subtotal (95% CI)**			**84**			**68**	**17.2%**	**0.00 [-1.12, 1.12]**
Heterogeneity: Not applicable								
Test for overall effect: Z = 0.00 (P = 1.00)								
**1.5.3 Moderate to severe**								
Ader 2021(DisCoVeRy) (3)	10	8.89	91	11	7.41	94	7.4%	-1.00 [-3.36, 1.36]
**Subtotal (95% CI)**			**91**			**94**	**7.4%**	**-1.00 [-3.36, 1.36]**
Heterogeneity: Not applicable								
Test for overall effect: Z = 0.83 (P = 0.41)								
**1.5.4 Severe**								
Ader 2021(DisCoVeRy) (4)	22	10.37	54	27	11.11	51	3.0%	-5.00 [-9.12, -0.88]
Alavi Darazam 2021(COVIFERON) (5)	5	4.03	40	7	3.31	20	9.9%	-2.00 [-3.91, -0.09]
Bhushan 2021 (6)	5.63	3.32	108	6.52	4.06	114	18.9%	-0.89 [-1.86, 0.08]
Davoudi-Monfared 2020 (7)	9.74	5.8	42	8.39	4.9	39	7.5%	1.35 [-0.98, 3.68]
Kalil 2021 (8)	5	4.89	368	5	4.97	380	22.3%	0.00 [-0.71, 0.71]
Rahmani 2020 (9)	9	2.96	33	11	4.44	33	10.5%	-2.00 [-3.82, -0.18]
**Subtotal (95% CI)**			**645**			**637**	**72.2%**	**-0.95 [-2.02, 0.12]**
Heterogeneity: Tau² = 0.99; Chi² = 14.47, df = 5 (P = 0.01); I² = 65%								
Test for overall effect: Z = 1.74 (P = 0.08)								
**Total (95% CI)**			**880**			**859**	**100.0%**	**-0.73 [-1.48, 0.02]**
Heterogeneity: Tau² = 0.53; Chi² = 15.51, df = 8 (P = 0.05); I² = 48%								
Test for overall effect: Z = 1.91 (P = 0.06)								
Test for subgroup differences: Chi² = 1.66, df = 3 (P = 0.65), I² = 0%								

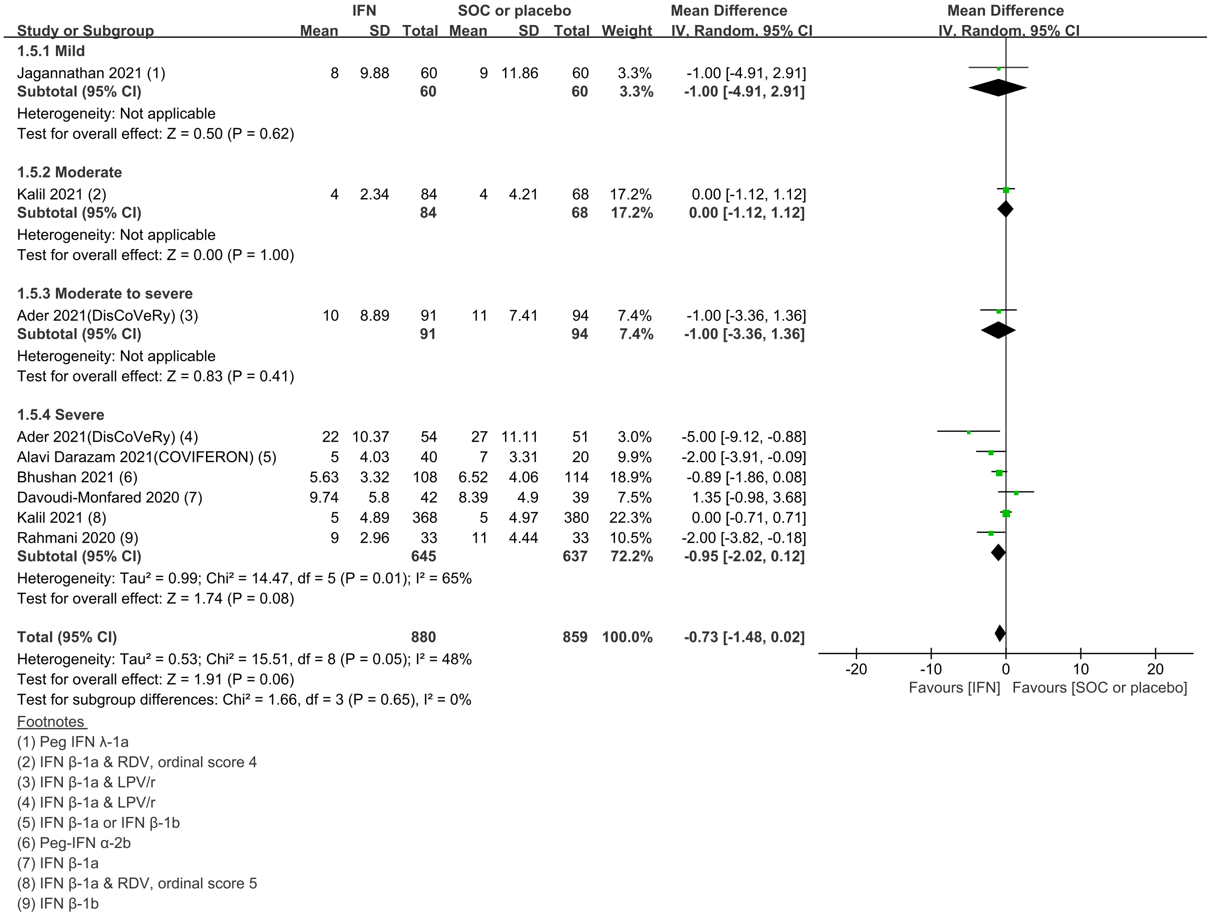

Footnotes

(1) Peg IFN λ-1a
(2) IFN β-1a & RDV, ordinal score 4
(3) IFN β-1a & LPV/r
(4) IFN β-1a & LPV/r
(5) IFN β-1a or IFN β-1b
(6) Peg-IFN α-2b
(7) IFN β-1a
(8) IFN β-1a & RDV, ordinal score 5
(9) IFN β-1b

S4 Fig. Forest plot of length of hospital stay

Study or Subgroup	IFN Mean	IFN SD	IFN Total	SOC or placebo Mean	SOC or placebo SD	SOC or placebo Total	Weight	Mean Difference IV, Random, 95% CI
**1.7.1 Moderate to severe**								
Ader 2021(DisCoVeRy) (1)	11	13.33	91	12	9.63	94	7.2%	-1.00 [-4.36, 2.36]
Kalil 2021 (2)	6	3.7	452	6	4.44	448	18.5%	0.00 [-0.53, 0.53]
**Subtotal (95% CI)**			**543**			**542**	**25.7%**	**-0.02 [-0.55, 0.50]**
Heterogeneity: Tau² = 0.00; Chi² = 0.33, df = 1 (P = 0.56); I² = 0%								
Test for overall effect: Z = 0.09 (P = 0.93)								
**1.7.2 Severe**								
Ader 2021(DisCoVeRy) (3)	29	0.74	54	29	3.7	51	16.6%	0.00 [-1.03, 1.03]
Alavi Darazam 2021(COVIFERON) (4)	5	2.76	40	6	1.48	20	16.5%	-1.00 [-2.07, 0.07]
Bhushan 2021 (5)	8.19	1.57	108	8.06	1.92	114	18.7%	0.13 [-0.33, 0.59]
Davoudi-Monfared 2020 (6)	14.8	8.45	42	12.3	7.48	39	6.9%	2.50 [-0.97, 5.97]
Kalil 2021 (7)	28	14.07	35	10	7.41	34	3.7%	18.00 [12.71, 23.29]
Rahmani 2020 (8)	11	2.96	33	13	5.19	33	11.9%	-2.00 [-4.04, 0.04]
**Subtotal (95% CI)**			**312**			**291**	**74.3%**	**1.14 [-0.65, 2.93]**
Heterogeneity: Tau² = 3.74; Chi² = 53.88, df = 5 (P < 0.00001); I² = 91%								
Test for overall effect: Z = 1.25 (P = 0.21)								
**Total (95% CI)**			**855**			**833**	**100.0%**	**0.40 [-0.74, 1.54]**
Heterogeneity: Tau² = 1.75; Chi² = 54.23, df = 7 (P < 0.00001); I² = 87%								
Test for overall effect: Z = 0.68 (P = 0.50)								
Test for subgroup differences: Chi² = 1.50, df = 1 (P = 0.22), I² = 33.3%								

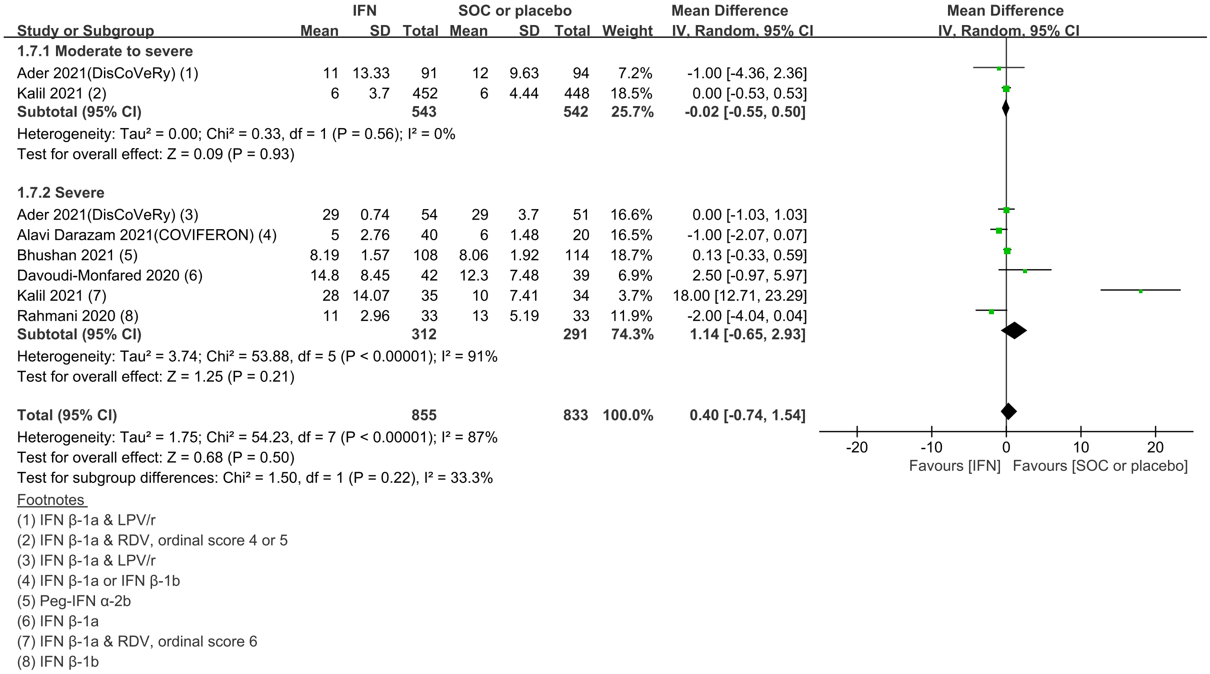

Footnotes
(1) IFN β-1a & LPV/r
(2) IFN β-1a & RDV, ordinal score 4 or 5
(3) IFN β-1a & LPV/r
(4) IFN β-1a or IFN β-1b
(5) Peg-IFN α-2b
(6) IFN β-1a
(7) IFN β-1a & RDV, ordinal score 6
(8) IFN β-1b

S5 Fig. Forest plot of hospitalization or emergency room visits in patients with mild COVID-19

	IFN		SOC or placebo			Risk Ratio
Study or Subgroup	Events	Total	Events	Total	Weight	M-H, Random, 95% CI
Feld 2021	2	30	5	30	39.7%	0.40 [0.08, 1.90]
Jagannathan 2021	7	60	5	60	60.3%	1.40 [0.47, 4.17]
**Total (95% CI)**		**90**		**90**	**100.0%**	**0.85 [0.26, 2.83]**
Total events	9		10			

Heterogeneity: Tau² = 0.32; Chi² = 1.67, df = 1 (P = 0.20); I² = 40%
Test for overall effect: Z = 0.26 (P = 0.79)

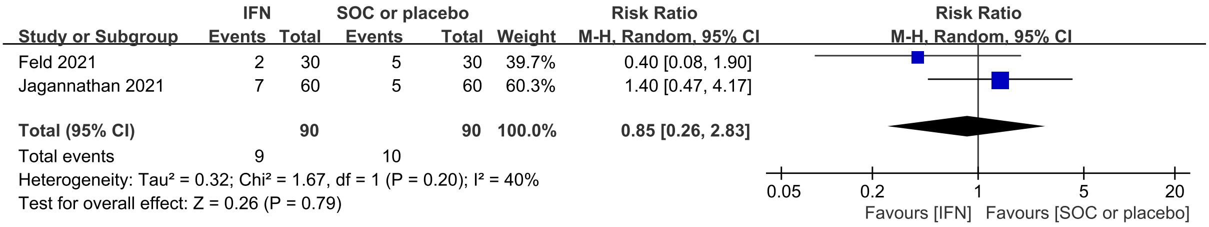

Supplement: S1 File — (PDF) [file pone.0272826.s002.pdf]
