# Supplementary material for: Clinical efficacy and safety of interferon (Type I and Type III) therapy in patients with COVID-19: A systematic review and meta-analysis of randomized controlled trials
Source: PLoS One. 2023 Mar 29;18(3):e0272826. doi: 10.1371/journal.pone.0272826 (PMC10057835; doi:10.1371/journal.pone.0272826)
Supplement: S3 File — (PDF) [file pone.0272826.s004.pdf]

	Random sequence generation (selection bias)	Allocation concealment (selection bias)	Blinding of participants and personnel (performance bias)	Blinding of outcome assessment (detection bias)	Incomplete outcome data (attrition bias)	Selective reporting (reporting bias)
Ader 2021 (DisCoVeRy)	+	+	?	?	+	+
Alavi Darazam 2021 (COVIFERON)	+	+	?	+	+	+
Bhushan 2021	+	?	?	?	+	+
Davoudi-Monfared 2020	+	+	?	?	−	+
Feld 2021	+	+	+	+	+	+
Jagannathan 2021	+	+	+	?	+	+
Kalil 2021	+	+	+	+	+	+
Monk 2021	+	+	+	+	+	+
Pan 2021 (Solidarity)	?	?	?	+	+	+
Pandit 2021	+	−	?	?	+	+
Rahmani 2020	+	+	?	+	−	+
