# Supplementary figures and images for: Clinical efficacy and safety of interferon (Type I and Type III) therapy in patients with COVID-19: A systematic review and meta-analysis of randomized controlled trials
Source: PLoS One. 2023 Mar 29;18(3):e0272826. doi: 10.1371/journal.pone.0272826 (PMC10057835; doi:10.1371/journal.pone.0272826)

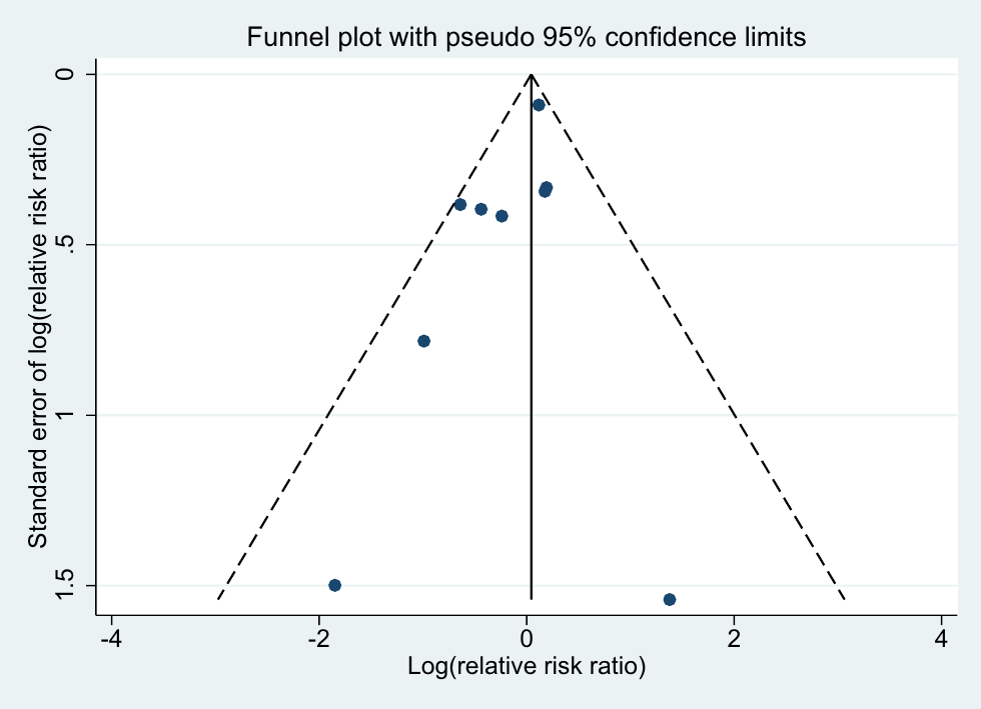

Supplement: S4 File — (TIF) [file pone.0272826.s005.tif]
